# Supplementary material for: CRISPR-induced indels and base editing using the Staphylococcus aureus Cas9 in potato
Source: PLoS One. 2020 Aug 17;15(8):e0235942. doi: 10.1371/journal.pone.0235942 (PMC7430721; doi:10.1371/journal.pone.0235942)
Supplement: S1 Fig — The reference sequence is displayed at the top of each panel, with the position of the PAM (in red, on the reverse strand) and the spacer sequences (in blue). The number on the right of each chromatogram corresponds to the number of identical chromatograms observed. The TCCA motifs that may explained the large deletion through a MMEJ repair pathway are underlined in purple. The Geneious software was used for the alignments. (DOCX) [file pone.0235942.s001.docx]

**S1 Fig1:** **Alignment of Sanger chromatograms obtained after TA cloning of individual PCR fragments of the StDMR6-1 targeted locus.** The reference sequence is displayed at the top of each panel, with the position of the PAM (in red, on the reverse strand) and the spacer sequences (in blue). The number on the right of each chromatogram corresponds to the number of identical chromatograms observed. The TCCA motifs that may explained the large deletion through a MMEJ repair pathway are underlined in purple. The Geneious software was used for the alignments.
