# Supplementary material for: CRISPR-induced indels and base editing using the Staphylococcus aureus Cas9 in potato
Source: PLoS One. 2020 Aug 17;15(8):e0235942. doi: 10.1371/journal.pone.0235942 (PMC7430721; doi:10.1371/journal.pone.0235942)
Supplement: S2 Fig — The SanCas9 sequence is in blue, the two NLS sequences in purple, the PmCDA1 sequence in green and the UGI sequence in red. All the coding sequence is optimized for expression in dicot species. (DOCX) [file pone.0235942.s002.docx]

ATGAAGAGGAACTACATCCTCGGACTCGCTATCGGAATCACCTCTGTGGGATACGGAATCATCGATTACGAGACTAGGGATGTGATCGATGCTGGTGTGAGACTCTTCAAAGAGGCTAACGTTGAGAACAACGAGGGAAGAAGGTCTAAGAGGGGAGCTAGAAGGCTCAAGAGAAGAAGAAGGCACAGAATCCAGAGGGTGAAGAAGCTCCTCTTCGATTACAACCTCCTCACCGATCACTCTGAGCTTTCTGGAATCAACCCTTACGAGGCAAGAGTGAAGGGACTCTCTCAGAAGTTGTCTGAGGAAGAGTTCTCTGCTGCTTTGCTCCACCTTGCTAAGAGAAGGGGTGTGCATAACGTGAACGAGGTGGAAGAGGATACCGGAAACGAGCTTTCTACCAAAGAGCAGATCTCTAGGAACTCTAAGGCTCTCGAAGAGAAGTACGTGGCAGAGCTTCAGCTTGAGAGACTCAAGAAAGATGGTGAGGTGAGGGGATCTATCAACAGGTTCAAGACCTCTGATTACGTGAAAGAAGCTAAGCAGCTCCTCAAGGTGCAGAAGGCTTACCATCAGCTCGATCAGTCTTTCATCGATACCTACATCGATCTTCTCGAGACTAGAAGGACCTACTACGAGGGACCTGGTGAAGGATCTCCATTCGGATGGAAGGATATCAAAGAATGGTACGAGATGCTCATGGGACACTGCACTTACTTCCCTGAAGAACTCAGATCTGTGAAGTACGCTTACAACGCTGATCTCTACAACGCTCTTAACGATCTCAACAACCTCGTGATCACCAGGGATGAGAACGAGAAGCTTGAGTACTACGAGAAGTTCCAGATCATCGAGAACGTGTTCAAGCAAAAGAAGAAGCCTACCCTCAAGCAGATCGCTAAAGAGATCCTTGTTAACGAGGAAGATATCAAGGGATACAGGGTGACCTCTACCGGAAAGCCTGAGTTCACTAACCTCAAGGTTTACCACGATATTAAGGATATTACCGCTAGAAAAGAGATTATTGAGAACGCTGAGCTTCTCGATCAAATCGCTAAGATCCTTACCATCTACCAGTCATCTGAGGATATCCAAGAGGAATTGACCAACCTCAACTCAGAGCTTACCCAAGAAGAGATCGAGCAGATCAGTAACTTGAAGGGTTACACCGGAACCCACAACCTCTCACTCAAGGCTATCAACCTCATCCTCGATGAGCTTTGGCACACCAACGATAACCAGATCGCAATCTTCAACAGACTCAAGCTCGTGCCTAAGAAAGTGGATCTCTCTCAGCAGAAAGAGATTCCTACCACCCTCGTGGATGATTTCATCCTCTCACCTGTTGTGAAGAGATCTTTCATCCAGTCTATCAAGGTGATCAACGCTATTATCAAGAAATACGGACTCCCTAACGATATCATCATCGAGTTGGCTAGGGAAAAGAACTCAAAGGATGCTCAAAAGATGATCAACGAGATGCAGAAGAGAAACAGGCAGACCAACGAGAGGATCGAAGAGATCATCAGGACCACCGGAAAAGAGAACGCTAAGTACCTCATTGAGAAGATCAAGCTCCACGATATGCAAGAGGGAAAGTGCCTCTACTCTCTCGAGGCTATCCCTCTTGAGGATCTCCTTAACAACCCTTTCAACTACGAAGTGGATCACATCATCCCTAGATCTGTGTCTTTCGATAACTCTTTCAACAACAAGGTGCTCGTGAAGCAAGAGGAAAACTCTAAGAAGGGTAACAGGACCCCTTTCCAGTACCTCTCTTCATCTGATTCTAAGATCTCATACGAGACTTTCAAGAAGCACATCCTCAACCTCGCTAAGGGAAAGGGTAGGATCTCTAAGACAAAGAAAGAGTACCTTCTCGAGGAAAGGGATATCAACAGATTCTCTGTTCAGAAGGATTTCATCAACAGAAACCTTGTGGATACCAGGTACGCTACCAGGGGTCTTATGAACCTTCTCAGGTCTTACTTCAGGGTGAACAACCTTGATGTGAAGGTGAAGTCAATCAACGGTGGATTCACCTCATTCCTTAGAAGAAAATGGAAGTTTAAGAAAGAGAGGAACAAGGGTTATAAGCACCACGCTGAGGATGCTCTCATTATCGCTAACGCAGATTTCATTTTCAAAGAGTGGAAGAAGTTGGATAAGGCTAAAAAGGTGATGGAAAACCAGATGTTCGAGGAAAAGCAGGCTGAGTCTATGCCTGAGATCGAGACTGAGCAAGAGTACAAAGAAATCTTCATCACCCCTCACCAGATCAAGCACATCAAAGATTTCAAGGATTACAAGTACTCTCACAGAGTGGATAAGAAGCCAAACAGAGAGCTTATCAACGATACCCTCTACTCAACCAGAAAGGATGATAAGGGAAACACCCTCATCGTGAACAATCTCAACGGACTCTACGATAAGGATAACGATAAGCTCAAGAAGCTCATTAACAAGTCTCCAGAGAAGTTGCTCATGTACCACCACGATCCTCAGACCTACCAAAAGCTCAAGCTCATCATGGAACAGTACGGTGATGAGAAGAACCCTCTCTACAAGTATTACGAGGAAACCGGTAACTACCTCACCAAGTACTCAAAAAAGGATAATGGACCTGTGATCAAGAAGATTAAGTACTACGGAAACAAGCTCAACGCACACCTCGATATCACCGATGATTACCCTAACAGTAGAAACAAGGTGGTGAAGCTTTCACTCAAGCCTTACAGGTTCGATGTGTACCTCGATAACGGTGTGTACAAGTTCGTGACCGTGAAGAACCTCGATGTTATCAAAAAAGAAAACTACTATGAGGTTAACTCTAAGTGCTACGAGGAAGCTAAGAAGTTGAAGAAGATCTCTAACCAGGCAGAGTTCATTGCTTCATTCTACAACAACGATCTTATCAAGATTAACGGTGAGCTTTACAGGGTTATCGGAGTGAACAACGATTTGCTCAACAGGATCGAGGTGAACATGATTGATATCACCTACAGGGAATACCTCGAGAATATGAACGATAAGAGGCCTCCTAGAATCATCAAGACTATCGCTTCTAAGACCCAGAGTATCAAGAAGTACTCTACCGATATCCTCGGAAACCTCTACGAGGTTAAGTCAAAGAAGCACCCTCAGATCATTAAGAAGGGATCAAGGGCTGATCCTAAGAAGAAGAGGAAGGTTGAATTCGGAGGAGGGCCAGGAGCAGAGTATGTTAGAGCGTTGTTTGATTTTAACGGTAACGATGAAGAGGATTTACCCTTTAAGAAAGGCGACATTCTCAGGATTAGGGATAAACCTGAAGAGCAATGGTGGAATGCTGAGGATAGTGAAGGCAAACGAGGAATGATTTTAGTGCCGTATGTGGAGAAATATTCGGGTGACTACAAAGATCATGATGGTGATTACAAAGACCATGACATCGACTACAAGGATGATGATGATAAGTCAGGGATGACAGATGCTGAATATGTCAGAATCCACGAAAAGTTGGACATTTACACGTTTAAGAAGCAGTTCTTCAACAACAAGAAATCTGTTTCGCATAGGTGCTATGTGCTTTTCGAACTAAAACGTCGTGGAGAAAGACGGGCTTGCTTTTGGGGTTACGCGGTTAACAAACCACAATCAGGTACTGAACGAGGAATACACGCTGAAATCTTTTCTATCCGAAAGGTTGAGGAATATCTACGTGACAATCCTGGACAGTTCACTATCAATTGGTATTCTAGCTGGTCACCATGTGCAGATTGTGCTGAGAAGATTCTCGAATGGTACAATCAAGAGCTTAGAGGCAATGGACATACATTGAAAATATGGGCATGCAAGCTCTACTACGAAAAGAATGCCAGAAACCAAATTGGGCTTTGGAACTTGAGGGATAATGGAGTTGGGCTTAATGTCATGGTTTCTGAGCACTATCAATGTTGTCGGAAGATCTTCATACAAAGTTCCCATAACCAGTTGAATGAGAACAGATGGTTAGAGAAAACCCTTAAAAGAGCCGAGAAGAGAAGATCCGAACTGAGCATTATGATACAGGTCAAAATTCTGCATACCACTAAGAGTCCAGCTGTAGGTCCTAAGAAGAAACGTAAAGTAGGGCCCATGACAAACCTATCAGACATCATTGAAAAAGAAACAGGTAAGCAACTCGTCATCCAGGAAAGTATCTTAATGCTCCCTGAAGAAGTCGAGGAGGTGATAGGCAATAAGCCTGAAAGTGACATACTTGTCCACACTGCCTATGATGAGTCTACAGACGAGAACGTCATGCTACTTACTTCAGACGCTCCAGAATACAAACCCTGGGCTCTTGTGATCCAAGATAGTAACGGAGAGAATAAAATAAAAATGCTGTGA

**S1 Fig 2: Coding sequence of the Sa-CBE developed in this study.** The SanCas9 sequence is in blue, the two NLS sequences in purple, the PmCDA1 sequence in green and the UGI sequence in red. All the coding sequence is optimized for expression in dicot species.
